# Home Telehealth Video Conferencing: Perceptions and Performance

**DOI:** 10.2196/mhealth.4666

**Published:** 2015-09-17

**Authors:** Alan Taylor, Greg Morris, Joanne Pech, Stuart Rechter, Colin Carati, Michael R Kidd

**Affiliations:** ^1^ Faculty of Medicine, Nursing and Health Sciences Flinders University Adelaide Australia

**Keywords:** telehealth, video conferencing, reliability, effectiveness, home care, mobile data networks, broadband

## Abstract

**Background:**

The Flinders Telehealth in the Home trial (FTH trial), conducted in South Australia, was an action research initiative to test and evaluate the inclusion of telehealth services and broadband access technologies for palliative care patients living in the community and home-based rehabilitation services for the elderly at home. Telehealth services at home were supported by video conferencing between a therapist, nurse or doctor, and a patient using the iPad tablet.

**Objective:**

The aims of this study are to identify which technical factors influence the quality of video conferencing in the home setting and to assess the impact of these factors on the clinical perceptions and acceptance of video conferencing for health care delivery into the home. Finally, we aim to identify any relationships between technical factors and clinical acceptance of this technology.

**Methods:**

An action research process developed several quantitative and qualitative procedures during the FTH trial to investigate technology performance and users perceptions of the technology including measurements of signal power, data transmission throughput, objective assessment of user perceptions of videoconference quality, and questionnaires administered to clinical users.

**Results:**

The effectiveness of telehealth was judged by clinicians as equivalent to or better than a home visit on 192 (71.6%, 192/268) occasions, and clinicians rated the experience of conducting a telehealth session compared with a home visit as equivalent or better in 90.3% (489/540) of the sessions. It was found that the quality of video conferencing when using a third generation mobile data service (3G) in comparison to broadband fiber-based services was concerning as 23.5% (220/936) of the calls failed during the telehealth sessions. The experimental field tests indicated that video conferencing audio and video quality was worse when using mobile data services compared with fiber to the home services. As well, statistically significant associations were found between audio/video quality and patient comfort with the technology as well as the clinician ratings for effectiveness of telehealth.

**Conclusions:**

These results showed that the quality of video conferencing when using 3G-based mobile data services instead of broadband fiber-based services was less due to failed calls, audio/ video jitter, and video pixilation during the telehealth sessions. Nevertheless, clinicians felt able to deliver effective services to patients at home using 3G-based mobile data services.

## Introduction

### Overview

The Flinders Telehealth in the Home trial (FTH trial) was conducted in South Australia during 2013 to 2014. The trial introduced telehealth services in community-based palliative care and home-based rehabilitation services for the elderly (Textbox 1).

For all groups, clinical teams emphasized the need for solutions appropriate for patients ≥65 years of age. The applications needed to be simple to use and interoperable with existing information and communications technology (ICT) infrastructure used by the health services. The equipment chosen was low-cost, consumer-grade, and as far as possible, based on non-proprietary standards.

**Services introduced during the Flinders Telehealth in the Home trial (FTH trial).**

Service

1. Community-based palliative care: Patients and their carers received video-conferencing and remote monitoring services from a palliative care nurse using a tablet device (eg, iPad), a self-assessment application to record their health status, and electronic devices and scales to monitor their physical activity and weight.
2. Home-based rehabilitation services for the elderly: Patients were remotely monitored by a therapist who made video calls as required [1]. They also had access to rehabilitation and speech therapists using a tablet device (eg, iPad), a self-assessment application to record their health status, and an exercise tracking device to monitor their physical activity.

### Technology

Clinical care was delivered from the Repatriation General Hospital, Adelaide, South Australia to participants using Internet Protocol-based video conferencing. Connectivity between the hospital and participants was achieved through the following mechanisms (1) the Australian National Broadband Network (NBN), a fixed line fiber to the premise network (FTTP) and (2) an Internet Service Provider (ISP) such as third and fourth generation (3G/4G) mobile data services (provided by a national telecommunications carrier (eg, Telstra).

**Figure 1:**
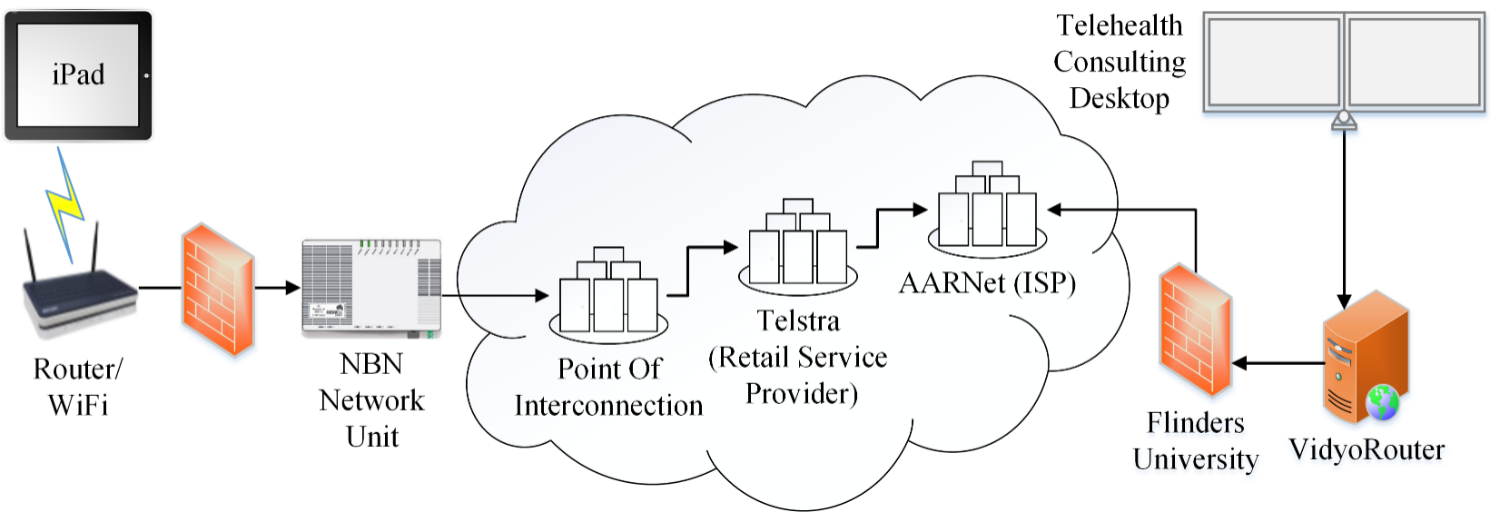
NBN network architecture.

**Figure 2:**
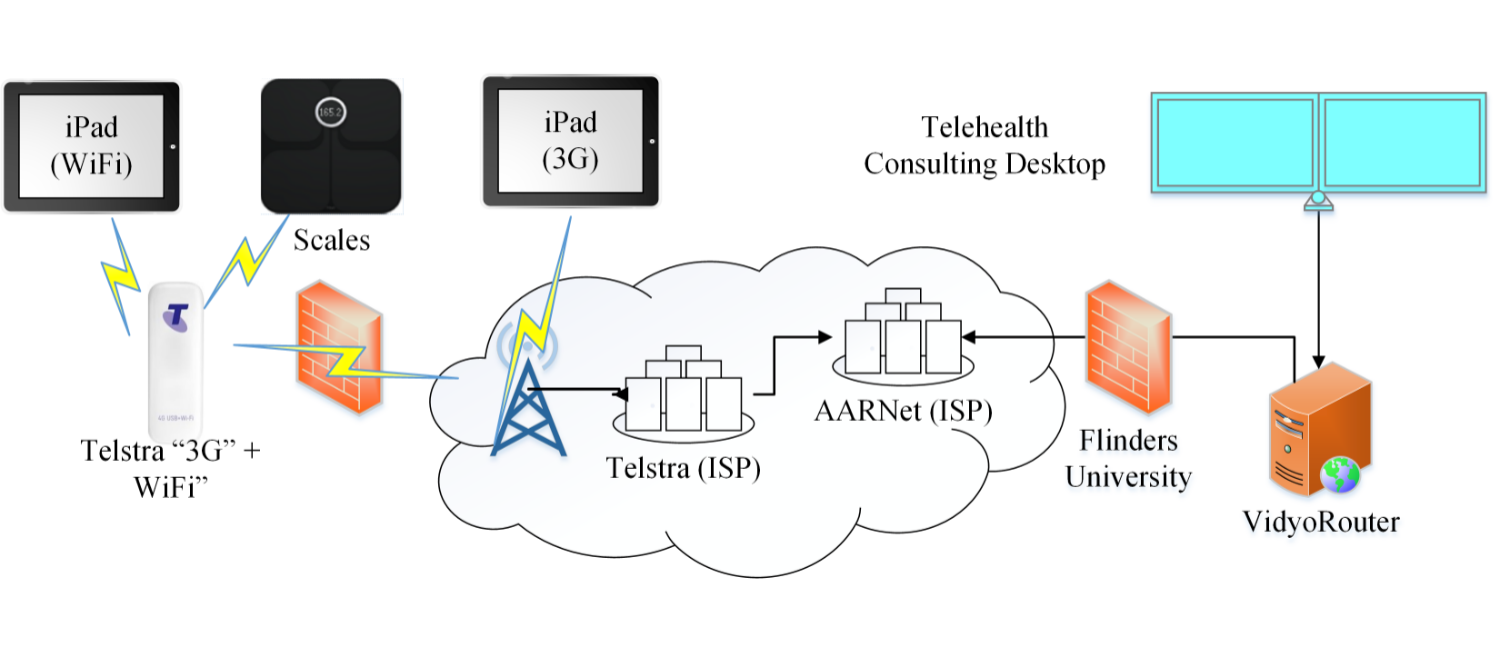
Mobile data network architecture.

Participants in their own homes were lent an Apple iPad tablet with WiFi connectivity to a NBN "ready" router with inbuilt wireless IEEE 802.11n (WiFi) and a NBN connection with a bitrate of 25 Mbps/5 Mbps (Figure 1) or an Apple iPad tablet with wireless connectivity through a standard commercial 3G/4G mobile plan (Figure 2). Palliative care patients were lent WiFi connected scales.

The video conferencing application used by the clinicians and FTH trial participants was based on a proprietary system Vidyo [2] hosted in the Flinders University data center to ensure low latency performance. Hospital-based clinicians scheduled calls to their patients using the Vidyo application. Clinicians conducted consultations from purpose built video consultation suites or iPads and participants answered video calls on their loaned iPads.

Prior work has identified the importance of technology factors on the success of home telehealth [3]. The research aims of our study are to identify which technical factors influence the quality of video conferencing in the home setting and to assess the impact of these factors on clinical perceptions and acceptance of video conferencing for health care delivery into the home.

## Methods

### Ethical Approval

An action research process developed several procedures during the FTH trial to investigate technology performance and users perceptions of the technology (Textbox 2).

Ethical approval was obtained through the Southern Adelaide Clinical Human Research Ethics Committee (HREC), application number HREC/13/SAC/88(168.13 and 203.13).

**Procedures developed during the Flinders Telehealth in the Home trial (FTH trial) investigation.**

Procedures

1. Simple tests of signal power, data download, and upload rates for Telstra mobile data 3G services (Telstra 3G) for differing received signal powers were undertaken at selected locations. Measurements of the signal powers for 4G services were not undertaken because in areas of poor coverage the equipment falls back to use 3G services.
2. Development of an experimental method to objectively measure the quality of audio, video, and failed calls in the field using Telstra 3G and FTTP services provided by the NBN.
3. Assessment of the reliability of video conferencing calls over the NBN and Telstra 3G using call log data from the Vidyo system to determine the average duration of calls, possible failed calls, and reconnection attempts.
4. A questionnaire for clinical video conferencing users of the NBN and Telstra 3G services to assess their experiences between the 3rd of March, 2013 and the 25th of July, 2014.

### Data Transmission Rates for Mobile Data Services

Due to the variable nature of radio propagation in the bands used by mobile data services it was difficult to validate Telstra 3G geographical coverage maps. In order to sample the real life performance of Telstra 3G services, a total of 249 measurements of the signal power levels (dBm) reported by the FieldTester (Staircase 3, Inc) application on the iPad tablet device [4], bitrates (kbps; up and down), and latency for a 64 Byte packet return trip reported by the Speedtest (by Ookla) application [5] were made at 14 locations using an iPad connected to the 3G network. Measurements provided a representative sample of high, medium, and low signal powers. The signal power at 10 second intervals was recorded for between 10 and 54 minutes at each location. Observations made during each minute were averaged to provide an indication of the signal power during each minute. Microsoft Excel software was used to analyze the results.

### Experimental Comparison of NBN and Telstra 3G services

**Figure 3:**
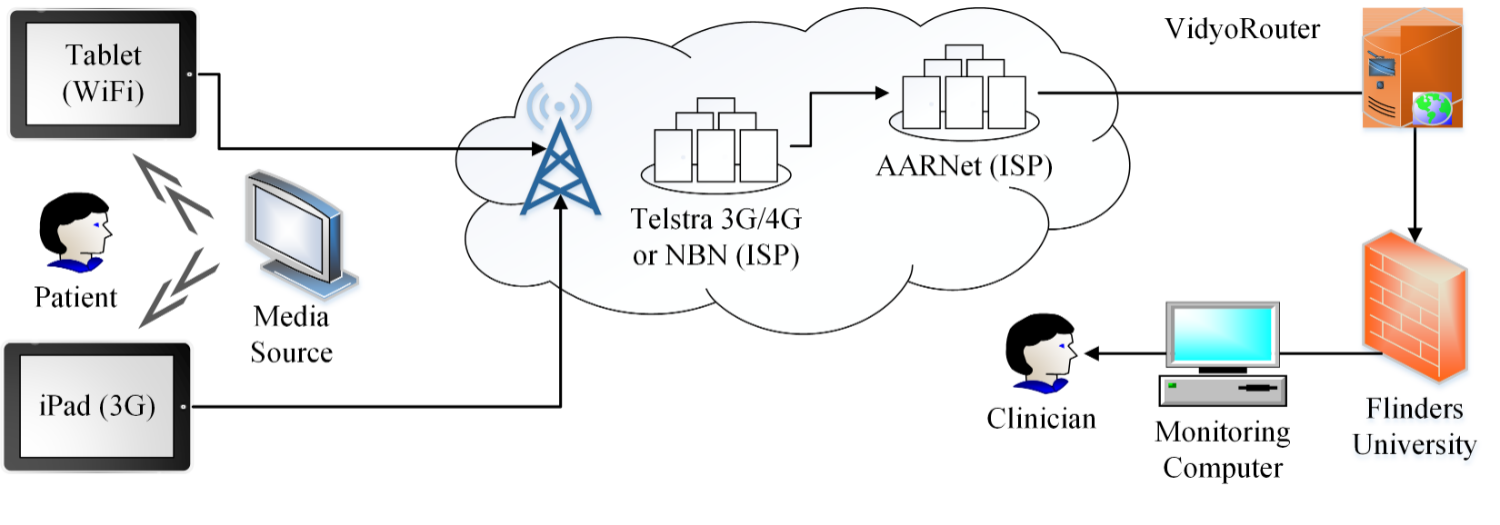
Experimental field tests setup.

To investigate the influence of using NBN compared with Telstra 3G networks on video conferencing an experimental method was developed to measure the quality of audio and video conferencing between a tablet device used by patients and a clinician’s computer. Each test involved a patient tablet that was placed in front of a media source which streamed the content to the clinician’s computer using a video conferencing client (Figure 3). As the log files provided by the Vidyo client did not provide network statistics related to video quality such as video packet loss, a staff member monitored each stream to determine the quality of the conference.

Clinical users felt that the key quality markers for a video conference were the number of times a call failed and had to be re-established, absent or delayed audio or video (jitter), and significant pixilation of the video. Using database software specifically developed to record the observations of the number and durations of negative events, each individual event (jitter and/or pixilation) observed was assigned with a start and end time. Failed calls were also counted. A failed call was a call that was unexpectedly terminated and included calls that were unable to complete a connection. While the results can be considered subjective, the same ICT staff member conducted all tests to minimize variances in the evaluation process.

Round trip Ping delay and signal power (dBm), as reported by the FieldTester application, were recorded using a second tablet at 10-second intervals; the begin and end timestamps of the other events were recorded with a one-second granularity throughout a call session time of 2700 seconds (approximately 45 minutes). A call session time of 45 minutes represented the average duration of a rehabilitation session. Average durations for palliative care were shorter (8 minutes).

Devices were configured to use Telstra 3G mobile data services only, because all 4G services will fall back to 3G services when the 4G signal power fades. This represented a more realistic telehealth conference as the targeted patient geographical locations were often in areas with poor 3G or 4G coverage. The different test combinations of broadband technology (Telstra 3G or NBN) and the different tablet devices that were tested are shown in Table 1. The tests were conducted over several days with a total duration of 58.11 hours using the Vidyo conferencing client.

**Table 1:** Test scenarios for networks, devices and test duration.

Broadband technology	Tablet connectivity	Tablet type	Duration, hours
NBN	WiFi to NBN router	iPad	9.85
3G	Telstra 3G SIM card	iPad	15.75
3G	WiFi to 3G access point	Samsung Galaxy	10.5
3G	WiFi to 3G access point	iPad	2.05
3G	3G SIM card	Android FonePad	8.25

### Reliability of Video Conferencing

An automated log of the calls made during the FTH trial using the Vidyo video conferencing system provided data for reliability analysis using the statistical software package SPSS. Call log records (N=4763) covered the period from October 20, 2013 to July 25, 2014.The call log contained data regarding the call duration, patient or clinician identifier and device identifier, the call start and end times, whether the call was via a conference room or direct, information on whether the call completed successfully or failed, and the call direction (incoming or outgoing). Data in the call log was cleansed of call records unconnected with clinical service (eg, test calls), and calls records were grouped to represent telehealth sessions when clinicians provided services to patients. A telehealth session comprised one or more calls and lasted between 5 to 45 minutes. If the first call to a patient failed for any reason, subsequent calls may have been made to re-establish the telehealth session. A total of 1021 (21.43%, 1021/4763) valid successful or unsuccessful telehealth sessions were identified.

### Clinical Perceptions of Video Conferencing

After each clinical consultation clinicians were asked to complete a computer-based questionnaire about the telehealth consultation that had just taken place. A total of 687 responses were received for consultations that used Telstra 3G and NBN technologies (Textbox 3).

**Computer-based questionnaire.**

Questions

- Clinicians’ perception of video and audio quality (1 very poor - 5 very good)

- Whether the patient appeared to be comfortable with the technology (1 not at all - 5 completely)
- Why was the patient uncomfortable with the video conference? (video/audio quality/connection issues/other)
- Rate and comment on the effectiveness of using telehealth with patients compared with home visit (1 much worse - 5 much better)
- Rate and comment on experience of using telehealth with patients compared to home visit (1 much worse - 5 much better)

To facilitate statistical analysis in SPSS, responses to questions about video and audio quality responses merged from 5 categories to 3. The “very good” responses were recoded as “good”. Similarly, the “very poor” responses were recoded to “poor”. Questions requesting further information as free text comments were graded for relevance to results of respondent's assessments using NVIVO 10 qualitative analysis software.

## Results

### Data Transmission Rates for Mobile Data Services

Clinical teams did encounter poor coverage in areas previously identified by analysis of Telstra 3G coverage maps. It was also observed that the location of the home and its construction could result in poor interior reception and that weather conditions were an additional factor. At one location signal power measurements (N=54) made during wet weather showed lower mean signal levels and greater variability than at the same location (N=32) during dry weather at −96.1 dBm (SD 8.3) and −86.7 dBm (SD 0.6), respectively. The analysis of all 249 measurements by signal power levels (weak 97 to −109 dBm; moderate −86 to -96 dBm, strong >−86 dBm) for data transmission rates, and latency in milliseconds is provided in Table 2.

**Table 2:** Signal power, data rates (kbps), and latency (milliseconds).

Signal power	Data rate down/up, kbps		Latency, mean (SD)
	Mean (SD)	Minimum	
Weak, N=70	4304/789 (4304/345)	72/39	96 (66)
Moderate, N=99	7376/1255 (3084/1001)	999/123	62 (18)
Strong, N=80	11077/2543 (4477/1542)	4477/1542	59 (26)

Most noticeable were the high variability of the upload (SD 0.61) and download (SD 0.56) rates, which in some cases, when signal powers were low, could reduce the minimum bitrates to values which could limit video and audio quality. At low power levels the average network latency rose to 96ms which may reduce the audio quality.

### Experimental Comparison of NBN and Telstra 3G services

**Figure 4:**
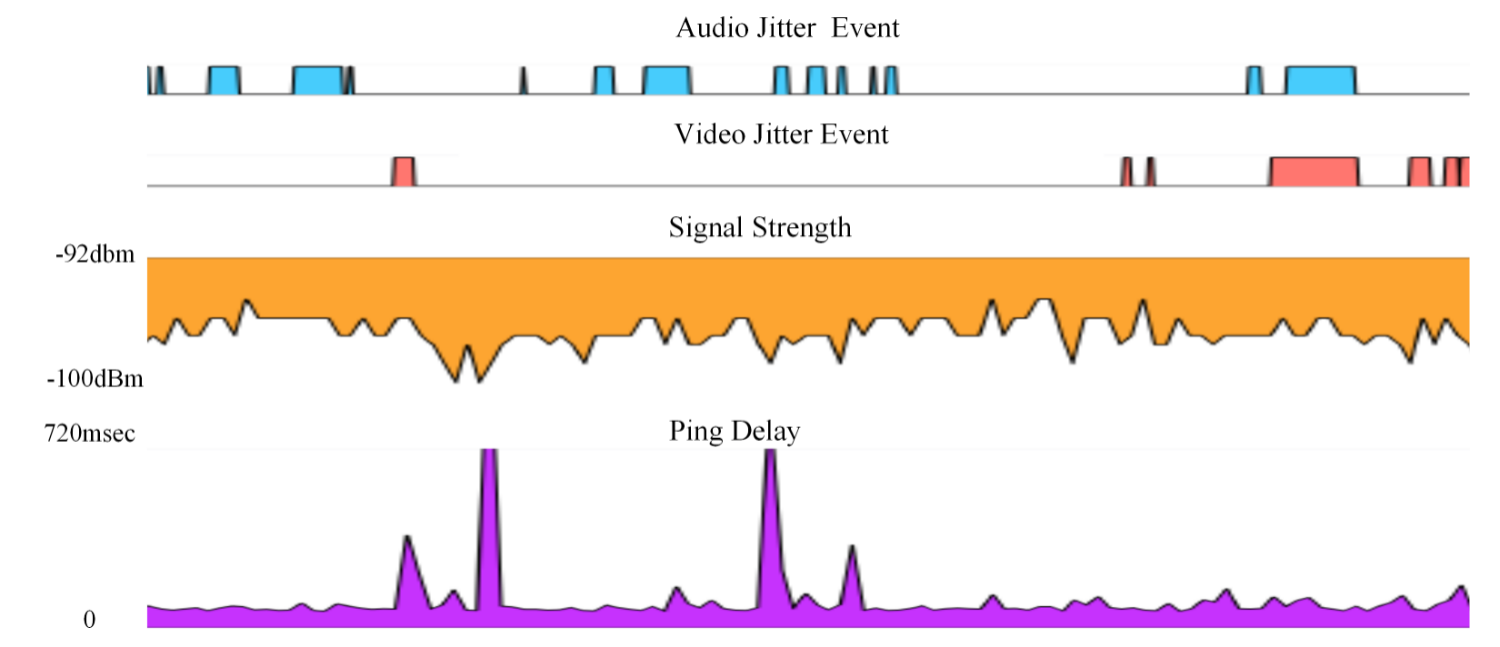
Typical recording of events, signal power and jitter.

The majority of the testing was undertaken when signal power was moderate but many instances of weak signal power were observed. Visual examination of 37 hours of experimental recordings of Telstra 3G signal power and events such as network packet "Ping" delay and/or audio and video jitter showed no time correlation between signal power and other events (Figure 4).

**Figure 5:**
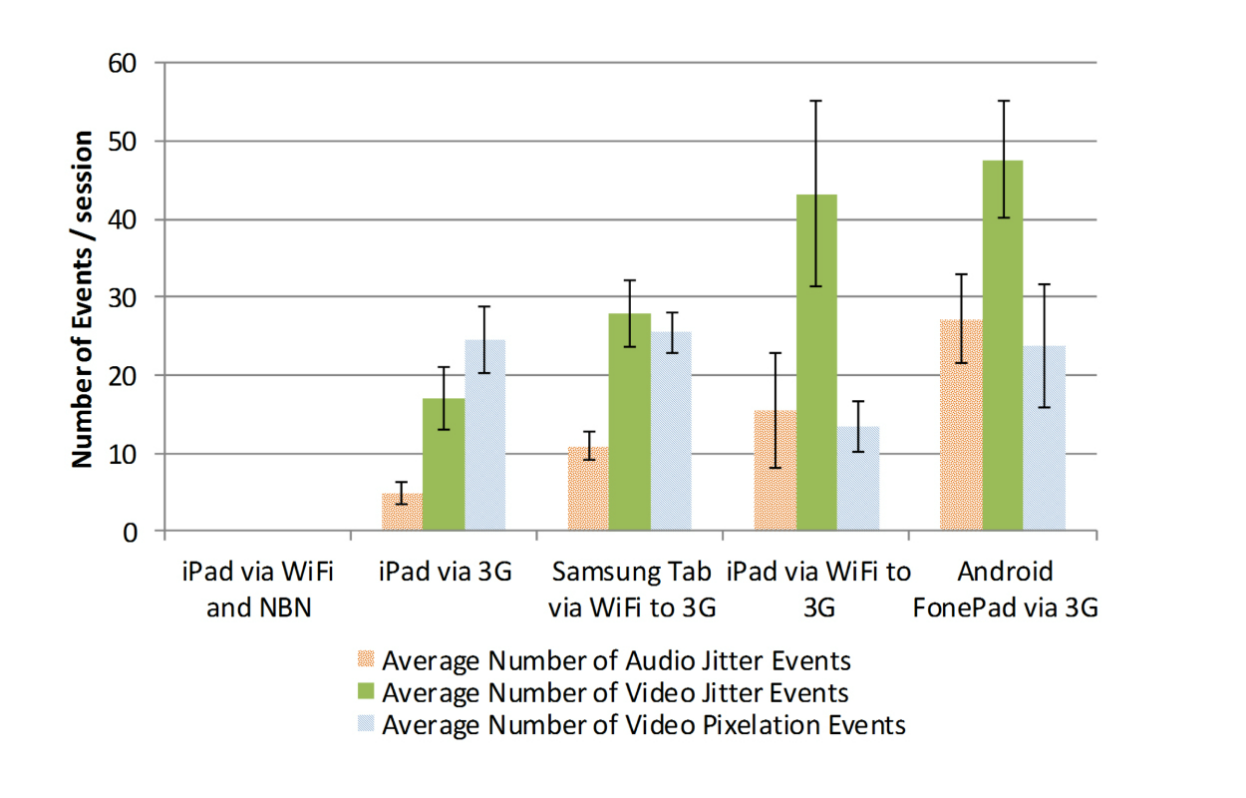
Comparison of Telstra 3G and NBN services (means and SE of session tests).

A comparison of the mean number of adverse events during each video conferencing session for each test combination shows the best performance was achieved by an iPad tablet via WiFi and NBN (Figure 5). While the rank order may change depending on the parameter chosen to rank each scenario (video and audio jitter or video pixilation events), the NBN-based scenario was always the best.

Across all test combinations, with the exception of those involving the NBN, the duration of the video, audio pixilation, and video jitter events varied from about 5 to 45 seconds with mean values between 15 and 20 seconds. Failed call events were of particular interest because these caused significant issues to telehealth sessions. Only one failed call was observed on the NBN. For the principal configuration deployed in the FTH trial (an iPad connected via Telstra 3G), 4 calls failed during 21 sessions lasting 45 minutes each. Differences between the performance of the iPad, Samsung Tab, and Android FonePad devices were observed for similar connections.

### Reliability of Video Conferencing

Of all the telehealth sessions identified in the call log, 936 (91.67%, 936/1021) were deemed successful. A successful session comprised one or more completed calls in the Vidyo call log. During the successful telehealth sessions, 220 (23.5%, 220/936) required more than one completed video call to be made by clinicians. There were 85 (8.33%, 85/1021) failed telehealth sessions due to more than one call terminating for any reason.

### Clinical Perceptions of Video Conferencing

A statistically significant positive association existed between clinical perceptions of video and audio quality (*P*<.001, Pearson chi-square coefficient 325.7). This was to be expected since both video and audio quality is related to the data transmission quality. In the clinician survey the quality of the audio and video for all types of connections was rated by clinicians as good for the majority of telehealth sessions. No statistically significant association existed between the NBN or Telstra 3G connection types and audio or video quality. Examples of clinician comments on audio, video, and reliability issues for the sessions that experienced technical difficulties are shown in Table 3.

**Table 3:** Examples of technical difficulties from the questionnaire responses (N=687).

Technical difficulty	Comments, n	Example quotes
Audio timing (echoes and delays)	22	"The audio was very poor today, the patient sounded like a robot ... it was very distorted and unclear, also very delayed".
Image quality	50	"Not crisp, but good enough for clinical purpose", or "Not receiving all frames so picture a bit jumpy" or "Not sharp but adequate".
Image timing (picture freezing, lack of synchronization with audio stream)	60	"Froze at least once every ten minutes" or "Froze x 1 however recovered" or "frozen picture, distorted, audio and visual not in sync, unable to continue therapy session"
Unsuccessful calls or reconnection attempts	22	"Patient unable to see and hear clinician for approx. 1 min (frozen), nil issues on clinician's end. Dropped out. No issues after re-dial" or "Regular freezing of picture requiring 3× hang ups and redials.

A statistically significant association was found between audio quality and patient comfort with the technology (*P*<.001, Pearson chi-square coefficient 95.3). When the patient was comfortable with the technology, audio quality was judged as good (86.3%, 403/467). When the patient was ambivalent, the audio quality was good in 67.6% (46/68) of the responses. When the patient was uncomfortable with the technology, the audio quality was poor in half (50.0%, 16/32) of the responses. An analysis of responses for different Internet types was not possible due to small sample sizes.

A statistically significant positive association existed between video quality and patient comfort with the technology (*P*<.001, Pearson chi-square coefficient 63.4). When the patient was comfortable with the technology, the video quality was rated as good in 404 (72.4%, 404/558) of the responses. Some patients were uncomfortable with the technology when initially learning how to use it (2.0%, 14/687), for example "couldn't turn machine on, then volume had been switched off". Some required ongoing supervision (2.3%, 16/687) possibly due to cognitive problems: "Continues to require phone call to open Vidyo prior to call".

Clinicians rated the effectiveness of conducting a session using telehealth compared with a home visit as equivalent or better 88.5% (478/540) of the time. A statistically significant association existed between audio quality and the effectiveness of conducting a session compared to a home visit using telehealth (*P*<.001, Pearson chi-square coefficient 142.1). The clinicians rated the audio quality as good on 444 (82.4%, 444/539) occasions. When clinicians rated the effectiveness of telehealth as equivalent to or better than a home visit, audio quality was reported as good in 313 (65.6%, 313/477) occasions. A statistically significant association also existed between video quality and the effectiveness of conducting a telehealth session compared with a home visit (*P*<.001, Pearson chi-square coefficient 96.4). When the telehealth effectiveness was rated as equivalent or better, video quality was reported as good in 368 (77.1%, 368/477) occasions.

Clinicians proved to be remarkably resilient in dealing with issues they encountered in using telehealth. Some significant issues did not prevent them from rating telehealth as effective or more effective than a home visit. For instance, out of 20 comments from clinicians rating telehealth as equivalent, one said "Interruption of video freezing impacted on the flow of the session". Another said "Relatively equivalent, some tasks unable to be completed however easily substituted with other tasks". A third stated:

Lost 20+ mins in establishing a connection. Session equivalent once connection established.

Clinicians rated the experience of conducting a session using telehealth compared with a home visit as equivalent or better 90.3% (489/540) of the time. Clinicians were also asked to comment on the how easy it was to provide a telehealth session compared to a home visit. Responses showed that on 71.6% (192/268) of the occasions, clinicians rated telehealth sessions as easier than a home visit focusing on time, travel, and efficiency savings (22 comments) as being key advantages. For example, one stated "Better as more efficient, achieved equivalent outcomes with no restrictions", and another said "No travel involved, often when you visit in person you may need to wait while the patient is finishing something else, but the patients' give priority to the videoconference". Clinical issues were cited by clinicians rating the telehealth session as less easy than a home visit. For instance, one said "abandoned session", and another:

More difficult this time. Previously ok when patient was non weight bearing and not allowed any ankle active movement. Now as weight bearing status has changed it was more difficult to perform my assessment and treatment.

##  Discussion

### Principal Findings

The main findings of this study are that the effectiveness and experience of home telehealth was judged by clinicians as equivalent to or better than a home visit and that the quality of video conferencing using 3G-based mobile data services in comparison to broadband FTTP services is less, due to failed calls during successful telehealth sessions, audio, video jitter, and video pixilation. However, clinicians were still able to deliver effective services to patients at home using this less than perfect technology.

Experimental field tests demonstrated significantly better performance of video conferencing over the NBN FTTP network, which provided an almost error-free performance for audio and video and no failed calls, whereas a greater proportion of adverse audio and video events were observed when using Telstra 3G mobile data connections. Recent work [6] has also reported that participants connected via 4G mobile data services experience more audio and visual difficulties than participants on the NBN. Earlier work [7] reported similar problems with 3G connectivity in areas of poor signal strength. Since NBN rollout has been slow and patchy, current (and likely future) telehealth services will continue to rely on 3G/4G mobile services. One recent study of videoconferencing over 4G networks found that 4G networks were an appropriate technology to deliver real-time video consultations; but due to known variability in performance of 4G networks, these (networks) should be evaluated prior to establishing a telemedicine service [8]. Other work has highlighted the value of understanding the actual mobile data coverage in urban New York in order to provide reliable services based on text messaging [9]. Laboratory-based tests discuss the performance of video conferencing used in emergency medical dispatch situations and report that video transmissions over a 3G network to a mobile phone froze for short periods during 21% of calls, which is consistent with our results [10,11]. Further research is required to determine if higher levels of reliability can be obtained using 4G services.

Measurements of data transmission rates over a Telstra 3G mobile data connection showed that 3G signal strength was a poor indicator of download or upload bitrates, and transmission rates can drop below levels needed to maintain good video and audio quality when the signal strength was weak or moderate. The extreme variability of transmission rates that can drop to less than half the average rate was a little surprising, and can only be the result of mobile data base stations and the associated backhaul network managing data rates to accommodate multiple users in real-time within an allocated base station capacity and other factors such as interference from other radio frequency sources or variable radio propagation due to weather related atmospheric layers. These findings support the reservations expressed by Holma that:

One common belief in the industry and among consumers is that a “faster network” provides a better user experience, meaning that a consumer’s experience with a smartphone will be inherently superior in an LTE network than an HSPA+/DC-HSDPA network. The reality can be somewhat different. [12]

12

It was anticipated that there could be an obvious correlation between intervals during which the 3G signal power was low and audio or video events. Further it was thought that there could be a correlation between intervals during which the "Ping" round trip delay time was high and the number/frequency of audio or video jitter events. In reality, even though large numbers of audio and video events occurred, there was no obvious time correlation observed between signal power and adverse audio, video or pixilation events, or the network delay time for 64 byte "Ping" packets.

Possible explanations for this behavior are that in a mobile data wireless environment the radio base station allocates resources continuously on a sub-second time scale. Data is transmitted in 2 ms or 10 ms blocks, and depending on signal power, interference or the presence of other users, modulation schemes and coding schemes may change from block to block [12]. These sub-second changes may reduce data throughput and increase packet delay. Video client decoding algorithms process video and audio packets on a sub-second time scale in order to manage delayed or missing data. Users simply see the combined results of this complex interaction. Degradations to audio and video quality are only observable when the information loss is so great that neither the network nor video client can compensate for the delayed or missing information. While fixed line Internet networks also experienced network transmission impairments such as congestion which could impact video and audio quality, significant network transmission problems were not observed on the NBN connection used in the experimental tests.

The relatively high ratio of failed calls to successful calls (compared to fixed and mobile telephony calls) made per telehealth session using Telstra 3G services is concerning. An average call failure rate of 23.5% during a telehealth session is high. By comparison, Telstra claims that the probability of a call failure will be less than 3% for mobile phone customers. We have no explanation for this application behavior. In theory, signaling messages between video conferencing clients require little network capacity, so calls should not fail even when video and audio quality is poor. In practice, calls do fail and fail to connect even when network capacity appears high. Despite the high failure rate of calls compared with the performance of calls on the mobile telephony network, clinical users appear to be willing to persist and try to make new calls to establish a telehealth session or re-establish a session if a call failed. On the whole, clinicians expressed satisfaction with the effectiveness of telehealth services in this health care setting even when using less than perfect telecommunications.

### Limitations

Many limitations to this study arose from the live, in-service nature of the research. Measurements of signal power depended on the FieldTester application for the iPad. This application has no publically traceable calibration of field strengths (volts/m) to signal power readings (dBm). Packet loss and packet jitter in networks can also significantly impact audio and video quality, but we were unable to find an application for tablet devices that would measure these parameters. Measurements of download and upload bitrates using the Ookla application were dependent on a pre-determined methodology over which we had no control. Finally it should be noted that sample sizes from the clinician questionnaire were insufficient to enable comparisons between the ways different clinical services perceived video conferencing technology.

###  Conclusions

Clinicians felt that the effectiveness and experience of home telehealth was equivalent to or better than a home visit even though the survey data indicated that audio and video quality during telehealth sessions was less than perfect. Clinicians persisted with using mobile data services even when calls failed and video or audio quality was degraded. We used a repeatable experimental method to measure video conferencing performance on mobile devices in the field and showed video conferencing services using 3G technology frequently suffer from failed calls, audio and video jitter, and video pixilation. Analysis of call logs also confirmed that failed calls were a frequent occurrence. As well, measurements of the data transmission capacity showed a high degree of variability, which may degrade video and audio quality.

The performance of tablet hardware and operating systems (Android and Apple IOS) differed, reinforcing the need to carefully select and test telehealth systems prior to deployment. While there was no significant association between the type of Internet connection used by the patient and audio or video quality as rated by clinicians, statistical analysis of clinical perceptions shows that the effectiveness of telehealth sessions and client comfort were closely linked to audio and video quality.

Field measurements for the video calls using the NBN demonstrated an almost error-free performance for audio and video. Given that 85.4% (587/ 683) of the telehealth sessions took place over mobile data services, it is reasonable to infer that the majority of audio and video quality issues experienced by clinicians could have been avoided if NBN connections had been available to participants in the project.

## Abbreviations

FTH trial: Flinders Telehealth in the Home trial
FTTP: fiber to the premises
ICT: information and communications technology
NBN: Australian National Broadband Network
3G/4G: third and fourth generation mobile data service
